# Prevalence-incidence bias in longitudinal studies of rheumatic heart disease in Fiji

**DOI:** 10.1186/s12872-025-05463-4

**Published:** 2025-12-23

**Authors:** Tuliana Cua, Shagorika Talukder, Mai Ling Perman, Daniel Engelman, Andrew Steer, Joseph Kado, Tom Parks

**Affiliations:** 1 Rheumatic Heart Disease Prevention and Control Programme, Fiji Islands Ministry of Health and Medical Services, Suva, Fiji; 2 Department of Infectious Disease, Imperial College London, London, UK; 3 Royal Papworth Hospital NHS Foundation Trust, Cambridge, UK; 4 Department of Internal Medicine, Fiji National University College of Medicine Nursing and Health Sciences, Suva, Fiji; 5 Infection, Immunity and Global Health, Murdoch Children’s Research Institute, Melbourne, Australia; 6 Wesfarmers Centre for Vaccines and Infectious Diseases, The Kids Institute, Perth, Australia; 7 Centre for Human Genetics, University of Oxford, Oxford, UK

**Keywords:** Rheumatic heart disease, Valvular heart disease, Epidemiology, Prevalence-incidence bias

## Abstract

**Background:**

Rheumatic heart disease (RHD), a consequence of a dysregulated immune response to *Streptococcus pyogenes* infection, remains the most commonly acquired cardiovascular condition in under 25-year-olds. Predominantly occurring in low- and middle-income countries (LMICs), RHD has an estimated prevalence of 40.5 million cases globally, although RHD complication rates in endemic settings remain uncertain. Accordingly, we undertook a prospective cohort study of RHD mortality and morbidity in Fiji with the aim of comparing complication rates to those estimated in our previous retrospective studies based on routine data and record-linkage from this setting.

**Methods:**

We prospectively ascertained RHD patients in the Central Division of Fiji from: (i) prevalent cases reviewed in echocardiography clinic prior to the study (May 2014-September 2015), and (ii) incident cases diagnosed in hospital (May 2014-October 2016). The primary endpoint comprised the earliest of RHD-attributable death, new onset heart failure or new onset stroke (assessed to October 2016). Differences between groups were assessed using a log-rank test (LRT). Additionally, to further investigate the issue of prevalence-incidence bias, rates of these events in the prospective cohort were also compared to those among patients with similar characteristics from our previously reported retrospective studies.

**Results:**

During a median of 19.3 months follow-up, the primary endpoint occurred in six out of a total of 163 patients recruited to the study, with an incidence rate of 1.8 (95% CI, 0.6–5.5) and 5.3 (95% CI, 1.7–16.5) per 100 person-years in the prevalent and incident groups respectively (LRT, *p* = 0.13). Among 617 comparable individuals from our previous retrospective studies, the primary endpoint occurred in 24 patients with an incidence of 1.4 (95% CI, 0.7–2.5) and 6.4 (95% CI, 3.8–10.8) per 100 person-years in prevalent and incident groups respectively (LRT, *p* = 0.002).

**Conclusions:**

Complications of RHD were less frequent in the prevalent compared to incident groups, reaching statistical significance in the larger retrospective cohort. Reflecting the impact of systematic differences between the groups, these findings highlight the vulnerability of longitudinal studies of RHD to prevalence-incidence bias, underscoring the need for further robust and representative studies of RHD outcomes in LMICs to inform efforts to limit the impact of RHD globally.

**Supplementary Information:**

The online version contains supplementary material available at 10.1186/s12872-025-05463-4.

## Background

Rheumatic heart disease (RHD), and its precursor acute rheumatic fever (ARF), a consequence of a dysregulated immune response to *Streptococcus pyogenes* (Group A Streptococcus, GAS) infection, remain the most commonly acquired cardiovascular condition in under 25-year-olds [1] and predominantly affects the young working population [2]. In 2022, the Global Burden of Disease study estimated that over 386,000 deaths and 13 million disability-adjusted life years (DALYs) were attributable to RHD, with 46.3 million prevalent cases of RHD globally [3]. In the largest contemporary cohort study to date as part of the INVICTUS trial registry involving 24 RHD-endemic LMICs and a total of 13 696 patients, the overall mortality rate was nearly 5% per year and higher in low income countries (LICs), reaching up to 7% [4].

Rheumatic heart disease is largely preventable, as evidenced by the low incidence and prevalence of RHD in high-income countries (HICs). However, to better tackle the global burden of disease, an improved picture of rates of death and disability is still needed [5]. This information is required not only to facilitate the many aspects of prevention [2], but also as a baseline for evaluation of the GAS vaccines that are currently under development [6].

The epidemiology of RHD in Fiji has been studied extensively [7–9]. Fiji is a middle-income country where RHD continues to be endemic and is a leading cause of death among young adults [8]. The drivers of this remain unclear but may include continued socioeconomic factors such as overcrowding and poor housing [7, 10], and repeated studies have found higher rates of disease and complications amongst the indigenous iTaukei population [9, 11]. We have previously used record-linkage to calculate nationwide, population-based estimates of RHD-attributable deaths [8], disability [9] and costs [12]. These data reveal a substantial burden of disease indicating RHD ranks among the leading causes of premature death in this setting [8]. Accordingly, given the inherent limitations of retrospective studies, we sought to refine and validate our retrospective estimates through a prospective cohort study.

The objective of our prospective cohort study was to measure the rates of complications of RHD among people living with RHD in the Central Division of Fiji, and to compare these to those estimated in our previous retrospective studies based on routine data and record linkage [8, 9]. Here we particularly explore the issue of prevalence-incidence bias, or ‘Neyman’s bias’, which impacts studies in which the onset of symptoms is associated with high case-fatality. Specifically, we sought to investigate the impact of this bias on longitudinal studies of RHD outcomes in Fiji where we have previously shown RHD complications are associated with substantially increased risk of death [9].

## Methods

### Study design

We undertook a prospective cohort study to measure rates of complications of RHD among people living with RHD in the Central Division of Fiji during 2014–2016. We also compared rates in the prospective cohort to those among patients with similar characteristics from our previously reported retrospective study [8, 9].

### Setting

Fiji is a middle-income country in the Western Pacific which at the time of the study had a population of approximately 900,000 individuals of which 43% reside in the Central Division [13]. Fiji is an island archipelago composed of more than 300 islands, but the majority of the population resides on the major islands of Viti Levu and Vanua Levu. The Central Division, situated on Viti Levu and containing the capital Suva, was our focus of patient recruitment, as only coverage of this area was feasible by a single research nurse. Inpatient and outpatient medical care for children and adults with RHD in the Central Division is provided at the Government-funded Colonial War Memorial Hospital (CWMH) in Suva [14].

### Prospective cohort

#### Patient selection

Patients were recruited to the study in two groups. The first comprised patients with *prevalent* RHD – individuals with the disease at the outset of the study – known to the national disease control programme who were reviewed in the echocardiography clinic at the Colonial War Memorial Hospital during 2011–2012. However, following a delay in initiating the study, these patients were not approached until May 2014 when the study commenced. The second comprised patients with *incident* RHD – individuals diagnosed with the disease during the study – not previously known to the national disease control programme newly presenting to the Colonial War Memorial Hospital up until October 2016. Patients were recruited by an experienced local clinical research nurse (TC). The prevalent patients were contacted in random order defined prior to the start of the study. Follow-up was at approximately six monthly intervals until the end of October 2016 (i.e. 30 months).

#### Endpoints

The primary endpoint comprised a composite of the earliest of RHD-attributable death, new onset heart failure and new onset stroke. Deaths were considered RHD-attributable if RHD (or valvular heart disease) was specifically mentioned as a cause on the death certificate, or where the cause of death was a recognised complication of RHD without an alternative explanation. Secondary endpoints were each of these events individually, infective endocarditis, cardiac surgery, adherence to secondary prophylaxis, and disability assessed by the New York Heart Association (NYHA) Functional Classification as well as the Modified Rankin Scales in patients with a history of stroke. Events were recorded using structured case report forms (CRFs) with online data entry by the research nurse. Potential risk factors for complications were also documented including comorbidity (e.g. type II diabetes mellitus).

#### Recruitment and follow-up

To increase ascertainment, the research nurse performed twice weekly rounds of the inpatient wards as well as daily rounds of the emergency department and the maternity hospital. In addition, patients (or their next-of-kin) were contacted by telephone, and where the research nurse was unable to make contact, with the assistance of local primary care nurses, visits to the patient’s home were made. More than two thirds of the cohort were reviewed on more than 3 separate occasions during the 1.5 years of the study. We obtained further information on uncontactable patients by monthly reviews of the electronic hospital patient information system, and on completion of the study we reviewed records of clinic attendance, admissions and deaths held by the Ministry of Health & Medical Services. The sample size was not pre-defined based on a power calculation, but for the prevalent group determined by an estimate of the number of cases that could be regularly reviewed by the study nurse.

### Retrospective cohort

For comparison, we selected individuals from our previously reported retrospective studies (8,9) covering the period 2008–2012. To maximise parity, the studies were compared based on the same primary endpoint based on diagnoses recorded in the patient information system [9]. Additionally, in the retrospective dataset, we limited analyses to 2011–2012, since underlying cause-of-death classifications were only available for this period [8]. For analysis, after excluding individuals subsequently recruited to the prospective cohort, we compared all individuals alive at the beginning of 2011 attending the echocardiography clinic during 2008–2010 (i.e. prevalent cases) to all individuals who had new hospital or outpatient attendance during 2011–2012 (i.e. incident cases). We excluded individuals with first hospitalisation for heart failure or stroke prior to 2011 for both groups.

### Statistical analysis

Data were inspected for missing values and outliers. There were no missing data for the variables used in this analysis. We calculated rates of death and complications using time to event analyses. Baseline events were defined separately to primary or secondary endpoints. In our primary analysis of the prospective cohort, we considered the interval from recruitment to the earliest of death to date or last review for the study. Additionally, as a sensitivity analysis, we extended the analysis period by 90 days for individuals whose electronic clinical record remained active up to the date of completion of the study (i.e. 21st October 2016). For the retrospective cohort, we considered the interval from, for the prevalent cases, the beginning of the analysis period (i.e. 1st January 2011), or, for the incident cases, the earliest recorded hospital or outpatient attendance, through to the earliest of death or end of the analysis period (i.e. 31st December 2012). Individuals with first hospitalisation for heart failure or stroke prior to or at the time of enrolment were excluded. As before, we assumed no loss to follow-up because we expected the patient information system would include the majority of hospitalisations during the study period. We performed a Cox multivariate regression analysis for the retrospective cohort to assess the effect of age, ethnicity and gender. The proportional-hazards assumption was checked after fitting the model on the basis of Schoenfeld residuals. Person-time in years was measured from the point of entry to the earliest occurrence of endpoint or exit from study. We used Kaplan-Meier curves to compare rates, and the log-rank test to assess for differences between groups. Analyses were performed in Stata (version 12).

### Ethical approval

The study was approved by the Fiji National Research Ethics Review Committee (FNRERC Number 2013-89) as well as the Oxford University Tropical Research Ethics Committee (1055-13).

## Results

### Recruitment and follow-up

**Fig. 1:**
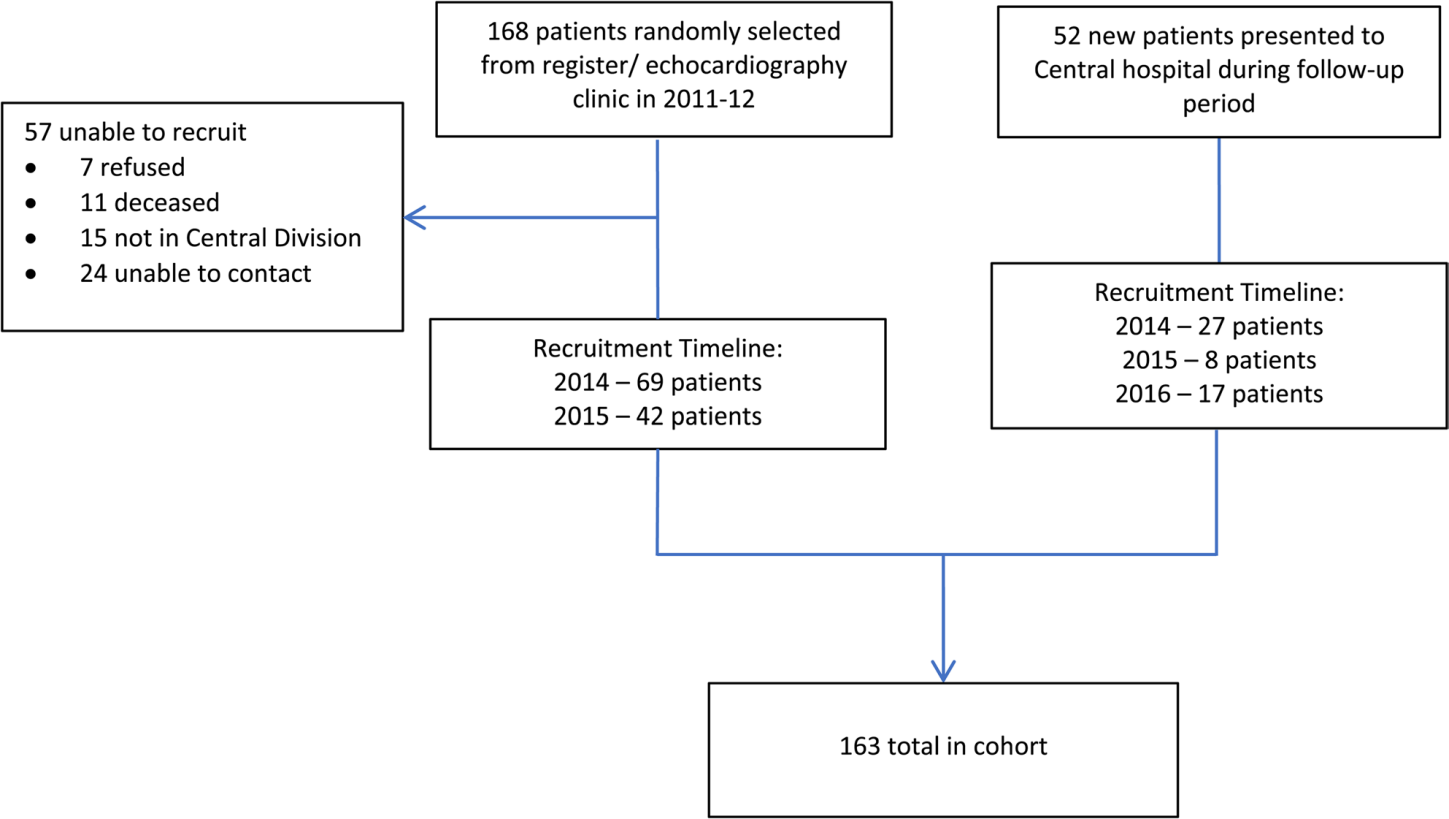
Recruitment and follow-up of the prospective cohort

Between May 2014 and September 2015, we recruited 111 (66.1%) of 168 prevalent RHD cases reviewed in the echocardiography clinic during 2011–2012. Of the remainder, seven (12.3%) refused to participate, 11 (19.3%) were deceased (at least six RHD-attributable), 15 (26.3%) did not live in the Central Division, and 24 (42.1%) could not be contacted (Fig. 1). We also recruited 52 incident cases newly presenting to the Colonial War Memorial Hospital before completion of follow-up in October 2016 of whom 33 individuals had been first diagnosed with RHD within the preceding 42 days and the remainder up to 20 months previously. The median follow-up period was 19.3 months (IQR 13.6–22.2) amounting to a median of three reviews (IQR 2–3) of each participant.

### Baseline characteristics of the prospective cohort

The combined prospective cohort of 163 individuals comprised a median age of 19.2 years (IQR 13.8–33.9) with only 17 (10.4%) aged more than 50 years (Fig. 1). More than half (62.0%) were female (Table 1). In the incident group, one individual was enrolled during hospitalisation for heart failure and one during hospitalisation for stroke. Additionally, three patients in the incident group had presented with features of ARF (two with carditis/polyarthritis and one with Sydenham’s chorea) while one patient in the prevalent group was enrolled during an episode of ARF recurrence (polyarthritis). Otherwise only a minority of the cohort reported cardiac symptoms, and these occurred almost exclusively in the incident group. There was a significant difference in the proportion of patients with NYHA Class II symptoms in the prevalent group compared to the incident group (1.8% vs. 9.6%, *p* = 0.03). Chest pain, fatigue and peripheral oedema occurred only in four in the incident group.

**Table 1:** Baseline characteristics of prospective and retrospective cohort

	Prospective cohort (*n* = 163)		Retrospective cohort (*n* = 617)		
	Prevalent (*n* = 111)	Incident (*n* = 52)	Prevalent (*n* = 379)	Incident (*n* = 238)	
Age in years – median (IQR)	20 (15–39)	16 (12–28)	21 (12–34)	22 (10 − 36)	
Female sex – n (%)	68 (61.3)	33 (63.5)	219 (58.1)	137 (58.1)	
Ethnicity:					
Indian descent – n (%)	22 (19.8)	3 (5.8)	108 (28.6)	66 (27.9)	
iTaukei / Rotuman – n (%)	88 (79.3)	45 (86.5)	226 (59.8)	155 (65.4)	
Other Pacific Islander – n (%)	1 (0.9)	4 (7.7)	44 (11.6)	16 (6.8)	
Past Events:					
Heart failure – n (%)	0	1 (1.9) †	‡	‡	
Stroke – n (%)	1 (0.9) †	2 (3.9) †	‡	‡	
Rheumatic fever – n (%)	0	1 (1.9)	*	*	
Prior cardiac surgery – n (%)	26 (23.4)	0	46 (12.1)	0	
Baseline events:					
Hospitalisation for heart failure – n (%)	0	1 (1.9) †	‡	‡	
Hospitalisation for stroke – n (%)	0	1 (1.9) †	‡	‡	
Rheumatic fever – n (%)	1 (0.9)	3 (5.8)			
Baseline symptoms:					
NYHA Class I – n (%)	109 (98.2)	47 (90.4)	-	-	
NYHA Class II – n (%)	2 (1.8)	5 (9.6)	-	-	
NYHA Class III-IV – n (%)	0	0	-	-	
Chest pain – n (%)	0	2 (3.8)	-	-	
Palpitations – n (%)	0	2 (3.8)	-	-	
Peripheral oedema – n (%)	0	1 (1.9)	-	-	
Fatigue – n (%)	0	3 (5.8)	-	-	
Baseline clinical echocardiography:					
Moderate mitral regurgitation – n (%)	30 (27.0)	10 (19.2)	-	-	
Severe mitral regurgitation – n (%)	9 (8.1)	10 (19.2)	-	-	
Moderate aortic regurgitation – n (%)	9 (8.1)	2 (3.9)	-	-	
Severe aortic regurgitation – n (%)	4 (3.6)	3 (5.8)	-	-	
Moderate mitral stenosis – n (%)	16 (14.4)	7 (13.5)	-	-	
Severe mitral stenosis – n (%)	13 (11.7)	7 (13.5)	-	-	
Baseline prophylaxis:					
BPG 3-weekly – n (%)	43 (38.7)	24 (46.2)	-	-	
BPG 4-weekly – n (%)	43 (38.7)	18 (34.6)	-	-	
Oral penicillin V – n (%)	2 (1.8)	3 (5.8)	-	-	
None – n (%)	23 (20.7)	7 (13.5)	-	-	
Other baseline medication:					
Warfarin – n (%)	30 (27.0)	4 (7.7)	-	-	
Aspirin – n (%)	4 (3.6)	2 (3.9)	-	-	
Heart failure therapy – n (%)	23 (20.7)	6 (11.5)	-	-	
Years since diagnosis – median (IQR)	7 (4–12)	0	-	-	
Occupation§:					
Employed – n (%)	17 (15.3)	7 (13.5)	-	-	
Full time education – n (%)	55 (49.5)	29 (55.8)	-	-	
Otherwise economically active – n (%)	2 (1.8)	1 (1.9)	-	-	
No occupation – n (%)	36 (32.4)	15 (28.9)	-	-	

*Rheumatic fever not reliably coded for retrospective cohort

†Individuals with past or baseline hospitalisation for heart failure or stroke prior to enrolment excluded from time to event analyses

‡Individuals with first hospitalisation for heart failure or stroke prior to 2011 excluded

§Data on occupation missing from one individual in the prevalent group

At baseline the proportion of individuals with moderate to severe mitral regurgitation (35.1% vs. 38.4%, *p* = 0.7), moderate to severe aortic regurgitation (11.7% vs. 9.6%, *p* = 0.5) and any mitral stenosis (31.5% vs. 36.5%, *p* = 0.6) on clinical echocardiogram was similar in the prevalent and incident groups. Similarly, the proportion with a mitral or aortic valve lesion of at least moderate severity was the same (54.1% vs. 59.6%, *p* = 0.6). Past complications of RHD were limited to one individual with a prior episode of ARF, one individual with prior heart failure and three individuals with past stroke. Comorbidity was limited to six individuals with one or more of ischaemic heart disease, hypertension, obesity or diabetes mellitus, and two individuals with other co-existent heart disease.

### Primary and secondary endpoints for prospective cohort

**Fig. 2:**
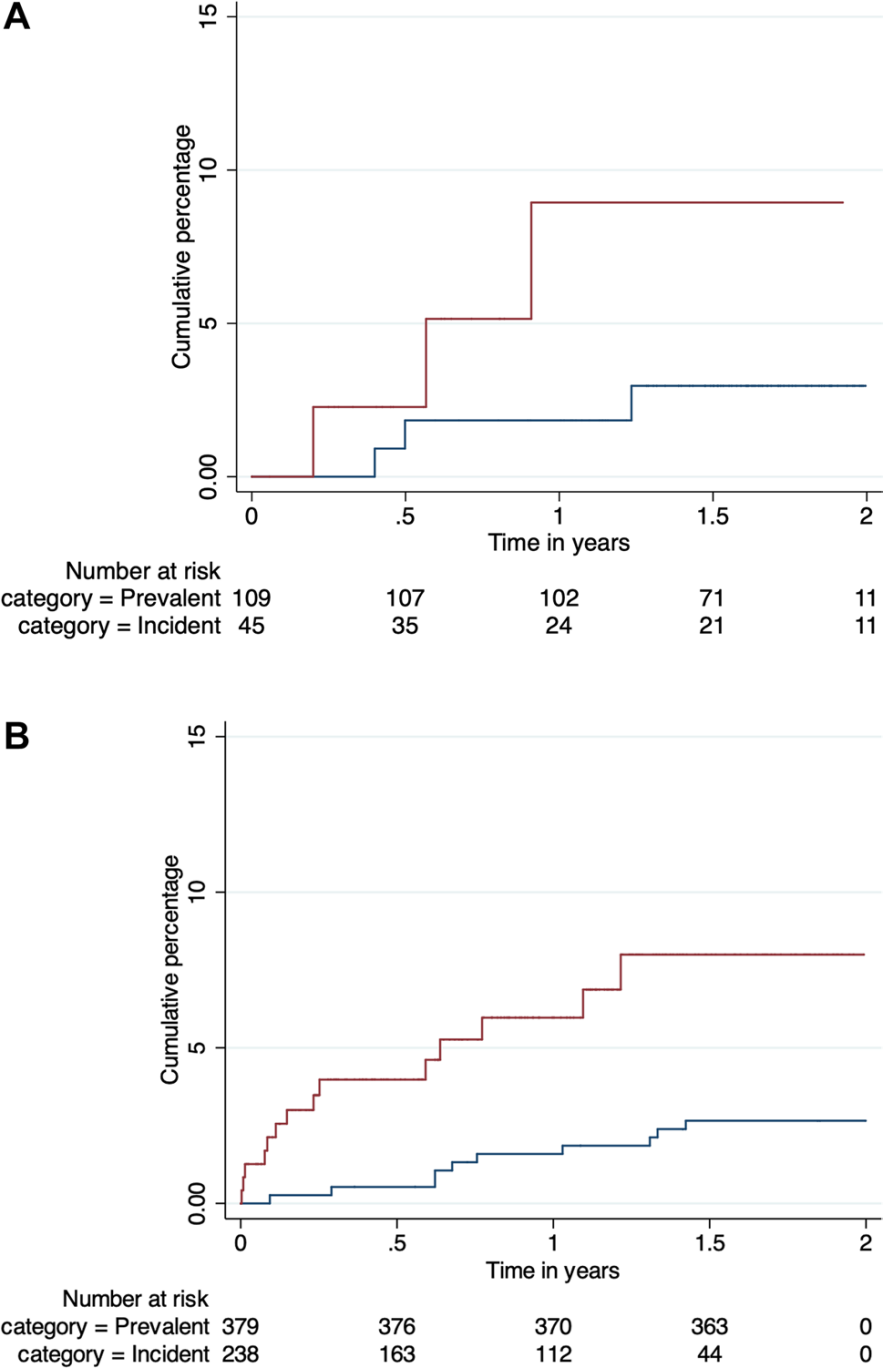
Cumulative percentage of the primary outcome among incident (maroon) and prevalent (blue) groups within (**A**) the prospective cohort and (**B**) the retrospective cohort. Plots show the Kaplan-Meier failure function as calculated in Stata together with the number at risk at six monthly intervals indicated in a table

Excluding six individuals with first hospitalisation for heart failure or stroke prior to or at the time of enrolment and three individuals without follow-up, the primary endpoint occurred in 3/109 (2.8%) of the prevalent group and 3/45 (6.6%) patients from the incident group (Table 2). This comprised one new stroke and four hospitalisations for new heart failure, one of which led to an RHD-attributable death. All but one event occurred before 40 years of age (range 11–60 years) with the RHD-attributable death occurring at age 39 years in the prevalent group. Four of the six events occurred in women, and all occurred in Indigenous iTaukei or Rotuman Fijians. All primary endpoint events occurred in individuals with a valve lesion of at least moderate severity at baseline. The overall incidence rates of these events combined was 2.7 (95% CI, 1.2–5.9) per 100 person-years across both incident and prevalent groups. However, the rate was 1.8 (95% CI, 0.6–5.5) and 5.3 (95% CI, 1.7–16.5) in the prevalent and incident groups respectively (Table 3), although this difference was not statistically significant by the log-rank test (*p* = 0.13) (Fig. 2A). Similarly, in the subset with at least moderate severity at baseline, the rate was 3.2 (95% CI, 1.0-9.9) in the prevalent group and 9.5 (95% CI, 3.1–29.4) in the incident group, although the difference was not statistically significant (*p* = 0.13). Further, findings were similar if individuals who had prior surgery were excluded and if the analysis period was extended by 90 days for patients with an active electronic clinical record to reduce bias in the incident cohort.

**Table 2:** Outcome events (primary outcome of earliest of RHD-attributable death, new onset heart failure and new onset stroke) during follow-up of prospective and retrospective cohorts

	Prospective cohort (*n* = 154)		Retrospective cohort (*n* = 617)			
	Prevalent (*n* = 109)	Incident (*n* = 45)	Prevalent (*n* = 379)		Incident (*n* = 238)	
Primary Outcome Event – n (%)	3 (2.8)	3 (6.6)	10 (2.6)		14 (5.9)	
RHD-attributable death – n (%)	1 (0.9)	0	4 (1.0)		4 (1.7)	
New onset heart failure – n (%)	3 (2.8)	2 (4.4)	*		*	
Hospitalisation for heart failure (first) – n (%)	3 (2.8)	1 (2.2)	5 (1.3)		9 (3.8)	
Hospitalisation for stroke (first) – n (%)	0	1 (2.2)	2 (0.5)		2 (0.8)	
Rheumatic fever recurrence – n (%)	2 (1.8)	0	†		†	
All cause death – n (%)	4 (3.7)	1 (2.2)	8 (2.1)		21 (8.8)	

*Heart failure without hospitalisation not reliably coded for retrospective cohort

†Rheumatic fever not reliably coded for retrospective cohort

**Table 3:** Event rates during follow-up of prospective and retrospective cohorts in 100 person-time years

	Prospective cohort (*n* = 154)		Retrospective cohort (*n* = 617)	
	Prevalent (*n* = 109)	Incident (*n* = 45)	Prevalent (*n* = 379)	Incident (*n* = 238)
Person-time – years	170.2	54.9	738.9	218.3
Primary outcome* – rate (95% CI)	1.8 (0.6–5.5)	5.3 (1.7–16.5)	1.4 (0.7–2.5)	6.4 (3.8–10.8)
Sensitivity analysis† – rate (95% CI)	1.6 (0.5–5.1)	5.0 (1.6–15.6)	-	-
RHD-attributable death – rate (95% CI)	0.6 (0.1–4.1)	0	0.5 (0.2–1.4)	1.8 (0.8–4.8)
New onset heart failure – rate (95% CI)	1.8 (0.6–5.5)	3.5 (0.9–13.8)	‡	‡
Heart failure hospitalisation – rate (95% CI)	1.8 (0.6–5.5)	1.7 (0.2–11.9)	0.7 (0.3–1.6)	4.1 (2.1–7.9)
Stroke hospitalisation – rate (95% CI)	0	1.7 (0.2–11.9)	0.3 (0.1–1.1)	0.9 (0.2–3.6)
ARF recurrence – rate (95% CI)	1.2 (0.3–4.7)	0	‡	‡
All cause death – rate (95% CI)	2.3 (0.9–6.2)	1.6 (0.2–11.7)	1.1 (0.5–2.1)	9.4 (6.2–14.5)

*Earliest of RHD-attributable death, new onset heart failure, and new onset stroke

†Analysis period extended by 90 days of individuals whose electronic clinical record remained active

‡Not reliably coded for retrospective cohort

Other events occurring during follow-up included five patients (3.1%) who underwent surgery, all comprising mitral valve surgery (+/- aortic or tricuspid valve surgery). There were four deaths unrelated to RHD. Nine acute admissions for heart failure among six patients occurred (in addition to two patients managed in outpatients), two for ARF, one for stroke and 12 for other diagnoses. There were no hospitalisations for infective endocarditis, valve thrombosis or systemic emboli. There were also five completed pregnancies with one emergency delivery and two by planned caesarean section, but no other maternal or neonatal complications.

### Comparison with retrospective cohort

We next compared the event rates in our prospective cohort with a subset of our previously described retrospective study re-analysed for the purposes of this study [9]. Specifically, after excluding 59 individuals subsequently recruited to the prospective cohort, we identified 617 individuals who had attended the echocardiography clinic during 2008–2012 of whom 379 (61.4%) and 238 (38.6%) were in the prevalent and incident groups respectively (Table 1). Differences with the prospective cohort included an older age profile and a greater proportion of Fijians of Indian descent in the incident group, and a lower rate of past cardiac surgery in the prevalent group.

There were eight RHD-attributable deaths, 14 heart failure hospitalisations and four stroke hospitalisations which together occurred at a median age of 29.4 years (IQR, 20.4–44.8) (Table 2). Combined, these events occurred at a rate of 1.4 (95% CI, 0.7–2.5) and 6.4 (95% CI, 3.8–10.8) per 100 person-years in prevalent and incident groups respectively (Table 3), which was statistically significant by a log-rank test (*p* = 0.002) (Fig. 2B). The difference between the incident and prevalent group remained apparent in a multivariate Cox regression model adjusting for age, gender, ethnicity and prior surgery (hazard ratio 3.4, 95% CI 1.4–8.0, *p* = 0.006) (Table 4).

**Table 4:** Hazard ratio estimates with 95% confidence intervals for risk of RHD-attributable death, first hospitalisation for heart failure or stroke in the retrospective cohort in a multivariate Cox regression analysis

Variable	Level	HR (95% CI)	*p* value
Study Group	Prevalent	1	
	Incident	3.4 (1.4-8.0)	0.006
Prior Cardiac Surgery	No surgery	1	
	Surgery	0.6 (0.1-5.0)	0.66
Sex	Male	1	
	Female	0.6 (0.2–1.3)	0.16
Ethnicity	iTaukei & others	1	
	Fijian of Indian Descent	0.6 (0.2–1.4)	0.21
Age (years)	5–14	1	
	15–39	2.0 (0.7–5.9)	0.18
	40–69	2.1 (0.6–7.3)	0.25

## Discussion

There is a clear need for more granular information about the impact of RHD in LMICs. In response, our data reassert the burden of complications in children and young adults in Fiji [8, 9, 11]. Nonetheless, the overall burden of complications estimated from our prospective cohort study was ostensibly lower than those reported in our previous retrospective studies based on record linkage [8, 9]. This prompted us to explore systematic differences in the population of patients reached by the two study designs. For example, the prospective cohort were somewhat younger and included a greater proportion of Indigenous iTaukei or Rotuman Fijians than the retrospective cohort. Nevertheless, we suspected a wider issue relating to our attempt to recruit patients living with prevalent RHD exacerbated by the delays in reaching this group, which consequently became a cohort of ‘survivors’. Crucially, even after excluding individuals with outcome events at the time of enrolment, this prevalent group, which made up more than two-thirds of the cohort, had a lower baseline proportion of patients with NYHA class II symptoms and a lower point estimate for the rate of the primary endpoint than the incident disease group. We replicated this pattern using a subset of our previous retrospective cohort selected to imitate the prospective cohort, where the difference in the primary endpoint rate between incident and prevalent groups reached statistical significance. Thus, the apparently lower rate of complications in our prospective cohort study reflects a study population enriched for survivors and illustrates the vulnerability of longitudinal studies of RHD to prevalence-incidence bias, a so far under-appreciated general feature of the epidemiology of the disease. A type of selection bias also termed ‘Neyman bias’, prevalence-incidence bias is expected where the onset of symptoms indicative of complications is associated with high case-fatality [15], and its impact on RHD epidemiology is analogous to that reported for other diseases such as pulmonary hypertension [16].

In our prospective study, the difference between incident and prevalent groups was exacerbated by an unavoidable delay in initiating recruitment of patients identified from echocardiography clinics. Unfortunately, the linked data from which these patients were selected were only available up to the close of 2012, and it was not possible to generate similar data for 2013 or early 2014. While this is undoubtedly a limitation of our study, it is important to stress the dominant model of RHD at the time that we conceived our study was one in which a proportion of patients living with prevalent RHD experienced long-term disability due to complication such as heart failure [1]. Additionally, studies of RHD are often based around registries, which naturally tend towards a prevalent patient population. In contrast, incident cases are often identified in hospitals once the disease is symptomatic and complications are apparent. Thus, the current study emphasises the need for further representative longitudinal studies of RHD using strategies such as pooling data from different sources combined by record-linkage. Indeed, in hindsight, the impact of prevalence-incidence bias is unsurprising given our Fijian studies showed clearly that the onset of RHD complications is associated with a marked shortening of life-expectancy [9].

Additional factors, including age, may also contribute to the lower complication rates observed in our prospective cohort, compared to the larger REMEDY cohort [17, 18] and other hospital-based studies [19–21]. For example, only 15% of our prospective cohort were aged over 40 years at recruitment compared to 26% of our retrospective cohort [9], and approximately 27% in the REMEDY cohort [18]. Similarly, our prospective cohort was substantially younger than the much larger INVICTUS registry in which higher complication rates were observed [4]. There may be additional differences between the retrospective and prospective groups that are unknown but even were this the case it remains likely that prevalence-incidence bias is a major contributor to event rates.

Regardless, it is important to emphasise that these are debilitating or fatal cardiac events occurring in children and young adults due to a preventable disease that now scarcely occurs in high-income settings. For example, five patients progressed to cardiac surgery for significant valvular disease and symptomatic RHD (with NYHA class III or IV breathlessness). Whilst this is fewer than in some contemporary studies, including the surgery rate of 15.3% reported from The Gambia [20] and 13.6% at baseline from the INVICTUS registry [4], these patients may represent the small proportion able to undergo surgery, relative to the total number eligible. The importance of valve surgery and mitral valvuloplasty is further emphasised by the recent cohort study from the INVICTUS registry demonstrating a ‘strong, independent, inverse association’ between intervention and mortality [4]. However, as within many LMICs, at the time of our study, cardiac surgery was only provided by visiting teams in Fiji. Furthermore, prevention of adverse outcomes in RHD also requires effective delivery of secondary prophylaxis, education of patients and their families, as well as advocacy and health promotion activities in the wider community [22].

Our prospective cohort had additional limitations including the small sample size and relatively short follow-up, which was determined by what could be achieved by our single research nurse with the funding available. The impact of this includes relatively imprecise estimates of rates, which may have meant we had insufficient power to detect a difference in event rates between incident and prevalent groups. We also had limited ability to detect rarer complications such as infective endocarditis and valve thrombosis. Additionally the sample size was not sufficient to perform the multivariate analysis that we applied to the retrospective cohort. Further work will provide additional information about the complication rates of RHD over a longer time-period, which currently is not available from most LMICs. We also had relatively limited echocardiographic data available, which was insufficient to investigate the impact of baseline cardiac function or other parameters on risk over time. Whether this information would explain differences between the incident and prevalent groups remains unclear, although it is notable that the proportion of individuals with moderate to severe valve lesions was similar in both groups. Furthermore, we had limited data available on socioeconomic determinants of health such as housing or education, and so were unable to assess their relationship to outcomes in this study. Moreover, we are unable to determine whether regular contact with a dedicated specialist research nurse – who was also tasked with identifying patients at risk of deterioration – had an impact on care of and outcomes of the cohort. Nonetheless, it is plausible that individuals in the cohort benefitted from this level of contact, including monitoring, education, and advocacy, all of which might have a knock-on effect on outcomes, as observed previously with the REMEDY study [23]. In reality, this was also a strength of our study, and the work of our research nurse (TC) allowed for detailed and high-quality data collection using a consistent methodology. Other strengths of our study included automated data entry checks with detailed review by local and international teams, and availability of death certificate data from the Ministry of Health and Medical Services. Finally, while we maintain that our findings continue to provide considerable value, we also recognise that due to a number of external factors several years have now passed since the period during which our study was conducted.

## Conclusions

In summary, our study represents one of very few prospective studies of RHD morbidity and mortality from an LMIC. It provides an important insight into the consequences of RHD in this population identifying important differences between incident and prevalent disease groups. Although our prospective study was limited to a single region of Fiji, our retrospective study covered the entire country. Accordingly, we anticipate that our observations may apply to other LMICs, at least in the Pacific region with a similar burden of RHD. More specifically, study designs that tend to recruit prevalent cases, who are more likely to be ‘survivors’, can expect to observe lower complication rates than designs recruiting incident cases with symptomatic disease. It provides important lessons for future studies of outcomes in other settings, emphasising the need to include people living with RHD from across the disease spectrum from both hospital and community settings in much-needed future research further describing the RHD morbidity and mortality in LMICs. Further such studies may allow for real-world advocacy and policy change to tackle this multi-factorial chronic condition. Given the marked concordance between the outcome findings of our prospective and retrospective studies, and the potential of the latter to reach a wider, more representative population of patients, robust retrospective studies based on real-world data are an essential first step and may be the force that drives the change.

## Supplementary Information

Supplementary Material 1.

Supplementary Material 2.

## Data Availability

Deidentified summary data from the prospective cohort underlying the time to event analyses are included in this published article and its supplementary information files. The primary dataset on which the retrospective cohort was based is the property of the Fiji Ministry of Health and Medical Services. Researchers wishing to access Fiji Ministry of Health data should send a request in writing to the Permanent Secretary of State for Health, Fiji Ministry of Health and Medical Services, 3rd Floor Dinem House, 88 Amy Street, PO Box 2223, Government Buildings, Suva, Fiji Islands (see also: http://www.health.gov.fj). Use of Fiji Ministry of Health data would also be subject to approval by the Fiji National Health Research Committee.

## References

[1] Watkins DA, Johnson CO, Colquhoun SM, Karthikeyan G, Beaton A, Bukhman G, et al. Global, Regional, and National burden of rheumatic heart Disease, 1990–2015. New Engl J Med. 2017;377:713–22. 28834488 10.1056/NEJMoa1603693

[2] Marijon E, Mocumbi A, Narayanan K, Jouven X, Celermajer DS. Persisting burden and challenges of rheumatic heart disease. Eur Hear J. 2021;42:3338–48. 10.1093/eurheartj/ehab407 34263296

[3] Mensah GA, Fuster V, Murray CJL, Roth GA, Global Burden of Cardiovascular Diseases and Risks Collaborators, Mensah GA et al. Global Burden of Cardiovascular Diseases and Risks, 1990–2022. J Am Coll Cardiol. 2023;82:2350–473. 10.1016/j.jacc.2023.11.007 PMC7615984 38092509

[4] Karthikeyan G, Ntsekhe M, Islam S, Rangarajan S, Avezum A, Benz A, et al. Mortality and morbidity in adults with rheumatic heart disease. JAMA. 2024;332:133–140. 10.1001/jama.2024.8258 PMC11154374 38837131

[5] Abouzeid M, Wyber R, Vincente SL, Sliwa K, Zühlke LJ, Mayosi B, et al. Time to tackle rheumatic heart disease: data needed to drive global policy dialogues. Glob Public Health. 2018;14:1–13. 30192707 10.1080/17441692.2018.1515970

[6] Walkinshaw DR, Wright MEE, Mullin AE, Excler J-L, Kim JH, Steer AC. The Streptococcus pyogenes vaccine landscape. Npj Vaccines. 2023;8:16. 36788225 10.1038/s41541-023-00609-x PMC9925938

[7] Steer AC, Kado J, Jenney AWJ, Batzloff M, Waqatakirewa L, Mulholland EK, et al. Acute rheumatic fever and rheumatic heart disease in fiji: prospective surveillance, 2005–2007. Med J Australia. 2009;190:133–5. 19203310 10.5694/j.1326-5377.2009.tb02312.x

[8] Parks T, Kado J, Miller AE, Ward B, Heenan R, Colquhoun SM, et al. Rheumatic heart Disease-Attributable mortality at ages 5–69 years in fiji: A Five-Year, National, Population-Based Record-Linkage cohort study. PLoS Negl Trop Dis. 2015;9:e0004033. 26371755 10.1371/journal.pntd.0004033 PMC4570761

[9] Parks T, Narube L, Perman ML, Sakumeni K, Fong JJ, Engelman D, et al. Population-based assessment of cardiovascular complications of rheumatic heart disease in fiji: a record-linkage analysis. BMJ Open. 2023;13:e070629. 37094887 10.1136/bmjopen-2022-070629 PMC10152053

[10] Dobson J, Colquhoun SM, Steer AC, Kado J. Environmental factors and rheumatic heart disease in Fiji. Pediatr Cardiol. 2012;33:332–6. 22057244 10.1007/s00246-011-0139-x

[11] Engelman D, Mataika RL, Kee MA, Donath S, Parks T, Colquhoun SM, et al. Clinical outcomes for young people with screening-detected and clinically-diagnosed rheumatic heart disease in Fiji. Int J Cardiol. 2017;240:422–7. 28400121 10.1016/j.ijcard.2017.04.004

[12] Heenan RC, Parks T, Bärnighausen T, Kado J, Bloom DE, Steer AC. The cost-of-illness due to rheumatic heart disease: National estimates for Fiji. T Roy Soc Trop Med H. 2020;114:483–91. 10.1093/trstmh/trz118 32232393

[13] Fiji Bureau of Statistics. Fiji population & housing census 2017. Fiji Bureau of Statistics. 2017. Available from: https://www.statsfiji.gov.fj/census-surveys/census-of-population-and-housing/. Accessed 1 Dec 2025. https://www.statsfiji.gov.fj/census-surveys/census-of-population-and-housing/.

[14] Roberts G, Irava W, Tuiketei T, Nadakuitavuki R, Otealagi S, Singh S, et al. Fiji Islands Health Syst Rev Health Syst Transition. 2011;1:1–150.

[15] Grimes DA, Schulz KF. Bias and causal associations in observational research. Lancet. 2002;359:248–52. 11812579 10.1016/S0140-6736(02)07451-2

[16] Miller DP, Gomberg-Maitland M, Humbert M. Survivor bias and risk assessment. Eur Respir J. 2012;40:530–2. 22941543 10.1183/09031936.00094112

[17] Karthikeyan G, Zühlke LJ, Engel M, Rangarajan S, Yusuf S, Teo K, et al. Rationale and design of a global rheumatic heart disease registry: the REMEDY study. Am Heart J. 2012;163:535–e401. 22520517 10.1016/j.ahj.2012.01.003 PMC5454482

[18] Zühlke L, Karthikeyan G, Engel ME, Rangarajan S, Mackie P, Mauff BC-K, et al. Clinical outcomes in 3343 children and adults with rheumatic heart disease from 14 Low- and Middle-Income countries. Circulation. 2016;134:1456–66. 27702773 10.1161/CIRCULATIONAHA.116.024769

[19] Makubi A, Hage C, Lwakatare J, Kisenge P, Makani J, Rydén L, et al. Contemporary aetiology, clinical characteristics and prognosis of adults with heart failure observed in a tertiary hospital in tanzania: the prospective Tanzania heart failure (TaHeF) study. Heart. 2014;100:1235. 24743164 10.1136/heartjnl-2014-305599 PMC5555386

[20] Jaiteh LES, Drammeh L, Anderson ST, Mendy J, Ceesay S, D’Alessandro U, et al. Rheumatic heart disease in the gambia: clinical and valvular aspects at presentation and evolution under penicillin prophylaxis. Bmc Cardiovasc Disor. 2021;21:503. 10.1186/s12872-021-02308-8 PMC8525010 34663206

[21] Desta TT, Gezachew A, Eshetu K. Descriptive analysis of rheumatic heart disease related complications in pediatric patients at tertiary Hospital, addis Ababa, Ethiopia. Pediatr heal. Med Ther. 2023;14:45–57. 10.2147/PHMT.S396854 PMC9930578 36817760

[22] Fiji ARF, RHD Guidelines Writing Group. Fiji guidelines for acute rheumatic fever and rheumatic heart disease Diagnosis, management and prevention: evidence based best practice guidelines. Suva, Fiji: Fiji Ministry of Health & Medical Services; 2017.

[23] Prendergast EA, Perkins S, Joachim A, Zühlke LJ, Mayosi B, Cupido B, et al. Participation in research improves overall patient management: insights from the global rheumatic heart disease registry (REMEDY). Cardiovasc J Afr. 2018;29:98–105. 29570206 10.5830/CVJA-2017-054 PMC6008904

